# Impact of a Novel Emergency Department Forward Treatment Area During the New York City COVID-19 Surge

**DOI:** 10.5811/westjem.2021.3.50653

**Published:** 2021-07-14

**Authors:** Joshua Brett Moskovitz, Kaushal Khambhati, Comilla Sasson, Jason D’Amore, Michael P. Jones, Jeremy Sperling

**Affiliations:** * Albert Einstein College of Medicine, Department of Emergency Medicine, Bronx, New York; † Hofstra School of Health and Human Services, Department of Public Health, Hempstead, New York; ‡ Albert Einstein College of Medicine, Jacobi Montefiore Emergency Medicine Residency, Bronx, New York; § American Heart Association, Dallas, Texas

## Abstract

**Introduction:**

Coronavirus disease 2019 (COVID-19) caused a disproportionate number of patients to seek emergency care at hospitals in New York City (NYC) during the initial crisis. Our urban emergency department (ED), a member of the NYC public hospital system had to process the increased volume while also differentiating our patients’ critical needs. We established a forward treatment area (FTA) directly in front of the ED to accomplish these goals from March 23–April 16, 2020.

**Methods:**

A clinical greeter evaluated patients 18 years and older who presented to the walk-in entrance of the ED where they were screened for COVID-19-like complaints. If they did not appear critically ill and could ambulate they were directed into the FTA. Clinical and non-clinical staff worked in concert to register, evaluate, and process patients with either a disposition of directly home or into the ED for further care.

**Results:**

A total of 634 patients were seen in the FTA from March 23–April 16, 2020. Of the 634 patients evaluated, 135 (21%) were referred into the ED for further evaluation, of whom 81 (12.7% of the total) were admitted. These patients were disproportionately male (91 into the ED and 63 admitted) and tended to have a higher heart rate (105.4 vs 93.7), a higher respiratory rate (21.5 vs 18.1), and lower oxygen saturation (93.9% vs 97.8%).

**Conclusion:**

A forward treatment area is an effective method to rapidly screen and process an increased volume of COVID-19 patients when resources are limited. This treatment area helped decompress the ED by being rapidly deployable and effectively screening patients for safe discharge home.

## INTRODUCTION

New York City (NYC) experienced a dramatic coronavirus disease 2019 (COVID-19) surge in March–April 2020. The Bronx, where our institution is located, was particularly overwhelmed, experiencing the highest hospitalization and mortality rates of the city’s five boroughs.^1^ This occurred despite the fact that the Bronx has the highest per capita number of hospital beds and the smallest number of elderly adults in NYC.^1^

Given the high demand for emergency care during the COVID-19 pandemic, many NYC emergency departments (ED) needed non-traditional methods to care for the influx of patients.

In prior disasters in the United States, such as Hurricane Katrina in 2005, the 2009 H1N1 influenza pandemic, and Hurricane Sandy in New York in 2012, alternative ED treatment areas had to be set up to handle patient surges. During the H1N1 pandemic, EDs used many options to meet the increased demand of patients. For example, in the state of Georgia, EDs created alternative treatment sites for high volumes of low-acuity patients in schools, community centers, mobile trailers, and outpatient clinics.^2^ Pediatric EDs in Texas used similar alternate treatment sites.^3^,^4^ After Hurricane Katrina and the closure of New Orleans’ Charity Hospital, a temporary ED was set up in a convention center and then in an abandoned department store; mobile clinics were also employed.^5^,^6^

Despite the many novel descriptions of alternative ED treatment areas, few, if any, reports describe the creation of an ad-hoc external ED treatment area to rapidly evaluate patients and preserve the functions of the existing ED. The literature focuses mainly on non-emergency sites distant from existing EDs (ambulatory care clinics, field hospitals, etc) or complementary treatment areas of the main ED offering a suite of services. Furthermore, there are limited descriptions of an ED forward treatment area (FTA) designed specifically to rapidly assess patients during an infectious disease outbreak.

During the COVID-19 surge, the volume of incoming patients with COVID-19-like symptoms rapidly overwhelmed the physical plant and resources of our ED. Daily ED volume increased by 20% with one third to one half of all patients presenting with COVID-19-like complaints. Although volume was only up 20%, the ED became rapidly overwhelmed because suspected COVID-19 patients required individual treatment rooms to avoid exposure to staff and other patients. Hallway stretchers and chairs, a commonality in NYC EDs during normal operations, had to be avoided. Rooms required more thorough cleaning between patients, and the ED needed to avoid crowding of patients in the waiting room. Patients with COVID-19-like complaints had remarkable variability in their illness severity, which necessitated rapid identification of patients requiring interventions from those who only needed education and reassurance.

With limited treatment options in the early stages of the pandemic and hospital space severely constrained, a new evaluation paradigm was required. Described herein are the characteristics of suspected COVID-19 patients cared for in an ED FTA, as well as factors associated with the need for further treatment in the main ED and/or admission to the hospital from March 23–April 16, 2020. Our primary objective in this study was to characterize the creation and methodology of an FTA as a means to decrease the throughput of the main ED. The secondary objective was to characterize the patients who passed through the FTA as well as their disposition.

## METHODS

This was a retrospective cohort study of all patients seen and evaluated in an ED FTA during the initial NYC COVID-19 surge from March 23–April 16, 2020. The primary outcome was the ability of the FTA to successfully and rapidly screen and discharge low-risk COVID-19 persons under investigation (PUI). The secondary outcome was to run an additional analysis on the clinical characteristics of screened patients with consideration for disposition outcomes (home vs requiring further evaluation in the main ED). The study was approved by the Albert Einstein College of Medicine Institutional Review Board.

**Population Health Research Capsule**

What do we already know about this issue?

*Little was known about ad-hoc processes to manage a sudden and sustained influx of infectious disease patients during a pandemic-level event*.

What was the research question?

*How can an emergency department (ED) use available resources to safely manage an infectious disease that is rapidly overwhelming resources?*

What was the major finding of the study?

*By establishing a forward treatment area (FTA) we were able to safely discharge 80% of patients without using main ED resources*.

How does this improve population health?

*The FTA saved scarce resources for patients who most needed them, allowing staff to concentrate on critically ill patients without significant adverse events*.

### Setting

Safety net urban hospital emergency department in the Bronx, NY, receiving >100,000 annual visits with a large emergency medicine residency program. The hospital is a Level I trauma center serving a limited- resource population; it is part of the nation’s largest public health hospital system.

### Description of Emergency Department Forward Treatment Area

**Figure 1:**
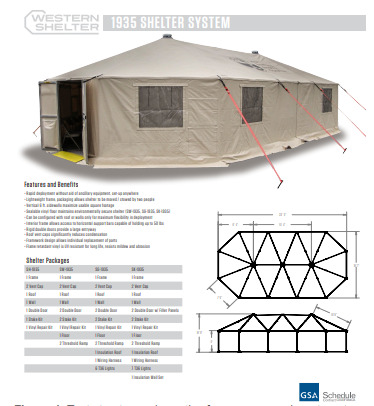
Tent structure schematics for emergency department forward treatment area.

**Figure 2:**
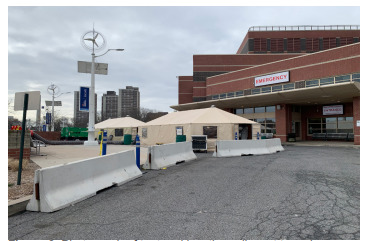
Photograph of tent and location adjacent to main emergency department.

**Figure 3:**
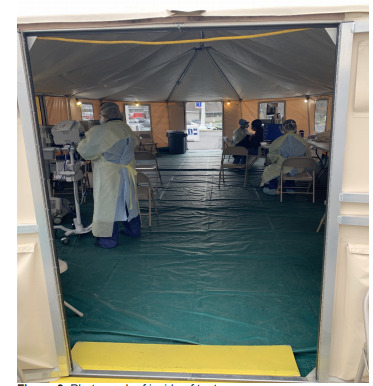
Photograph of inside of tent.

The ED FTA was comprised of two tents provided and set up by the NYC Office of Emergency Management (OEM) (Figure 1). The tents were placed directly outside (within 15 feet) of the main ambulatory ED entrance (waiting room) and labeled “COVID screening area” (Figure 2). The tents were equipped as delivered and installed by OEM with full power for lighting, computers, and heating, ventilation and air conditioning. The ED provided and installed seating and screens (to keep patients physically distanced >6 feet apart); Wi-Fi access points; workstations on wheels (WOW) with full electronic health record access and integration in each tent; printers; vital sign units for recording full sets of vital signs; and hand hygiene stations (Figure 3).

### Forward Treatment Area Workflow and Protocols

**Figure 4:**
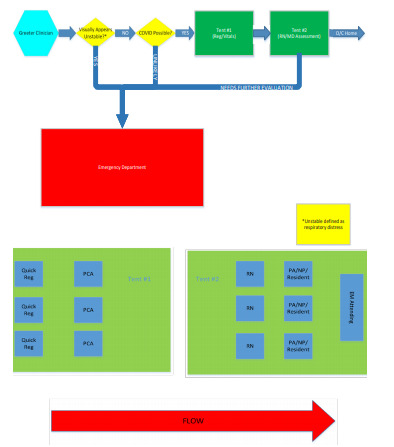
Forward treatment area pathway.

Outside the main ED entrance, a “greeter” (Registered Nurse (RN) or Physician Assistant (PA)) met every patient at the walk-in entrance and visually assessed them (Figure 4). The visual assessment focused on the greeter’s subjective assessment of respiratory distress (obvious distress/ability to speak in full sentences). If they appeared unstable, they were immediately brought inside the main ED for standard ED triage. If the patients appeared stable, they were assessed for reason of visit, and if related to COVID-19 subjectively (broad capture of symptoms such as fever, shortness of breath, cough, nausea / vomiting / diarrhea), they proceeded through the FTA. As the environment outside was winter in the Northeast the greeter’s assessment was entirely visual and brief although the predominance of patients presenting at the time were of a COVID like nature. Patients were excluded from the area and seen directly in the ED if they were less than 18 years of age or were unable to ambulate independently or were presenting to the ED for primarily non-COVID-19 related complaints (eg, suture removal).

No testing for COVID-19 was done in the FTA, in compliance with the recommendations of the NYC DOH, because testing samples were in extremely short supply at that time.

Patients flowed unidirectionally through the FTA as follows:

1. Clerical staff registered the patient;
2. A patient care associate (PCA) obtained vital signs;
3. An RN performed rapid assessment and completed abridged triage;
4. A resident physician, PA, or nurse practitioner (NP) completed rapid evaluation and presented to the ED attending;
5. The attending emergency physician oversaw evaluation and disposition decision.

All members of the FTA with the exception of the clerical staff and the ED Attending did not necessarily have emergency medicine experience. These team members came from other departments within our hospital as well as volunteers and locums (RN, PA, MD) brought in by the health care system. The team members from other hospital departments included RN administrators, NPs, and residents (dental, pediatrics, etc.). The team members received Just-In-Time (JIT) Training (designed ad-hoc) at minimum of two times: on appointment to the team which included a broad overview prior to the initiation of the FTA and every morning when reporting for duty during an 0800 hour ED-wide huddle including a separate more focused second huddle prior to entering the FTA (see Appendix 1). The greeters had no specific additional training or experience. The final disposition diagnosis and decision was made by the ED Attending physician present in the FTA utilizing clinical judgement as no scoring systems existed at the time and the disease was relatively unknown.

The RNs performing the rapid assessment, the intermediary assessment by the resident/PA/NP, and the attending emergency physician were all within close proximity to one another allowing sharing of information and co-assessments adjusting for volume. Up to three lanes of RN and intermediary assessment could be performed at one time depending on patient volumes with the EM attending overseeing the processes.

The FTA operated approximately 11 hours daily (9am to 8pm). This was set to start the shift with the 0800 hours ED huddle and coincide with previously established twelve-hour shift rotations.

### COVID-19 Electronic Health Record SmartSet

To expedite throughput, team members used a COVID-specific SmartSet that included a prepopulated note template, diagnosis and prepopulated discharge instructions.in the EHR (Epic Systems Corporation, Verona, WI). A medical screening evaluation, fully compliant with the Emergency Medical Treatment and Labor Act, could be completed in fewer than three minutes with personalized printed discharge instructions. This Epic SmartSet was designed by our centralized hospital system. When triaged in the FTA the patient was also flagged as a “mass influx” patient within Epic and roomed in the “disaster” treatment area.

### Personal Protective Equipment

Staff were required to wear full, “level 1” personal protective equipment (PPE) described by the New York City Health and Hospitals Corporation (NYCHHC) at that time as follows: N95 mask covered by a surgical mask; goggles; hair cover; double gloves; and surgical gown. Between patients, all staff changed outer gloves and sanitized their hands with an alcohol-based solution. Power air-purifying respirators were not available. During this initial surge PPE supplies were not abundant.

### Data Collection

Demographics, initial vital signs, and clinical dispositions were collected for all patients presenting to the FTA area between March 23–April 16, 2020. To capture any return ED visits after evaluation in the FTA, outcomes were collected for an additional two weeks after study conclusion.

### Data Analysis

We report descriptive statistics for continuous variables as means with interquartile ranges (IQR). Categorical variables are reported as counts and percentages. We divided patients into three categories: discharged from the FTA (n = 499); further assessed in the ED (n = 135); and admitted (n = 81). We used multivariable logistic regression to model the associations between needing additional evaluation in the ED or being admitted, with age, gender, initial vitals (pulse, respiratory rate, and oxygen saturation) as payer categories. To assess the quality of the model for goodness of fit we used Hosmer-Lemeshow statistics in Stata v13.0 (StataCorp, College Station, TX). We further assessed for a safety endpoint of a potential incorrect disposition, which we defined a priori as a discharge followed by a mortality in the following seven days.

## RESULTS

**Figure 5:**
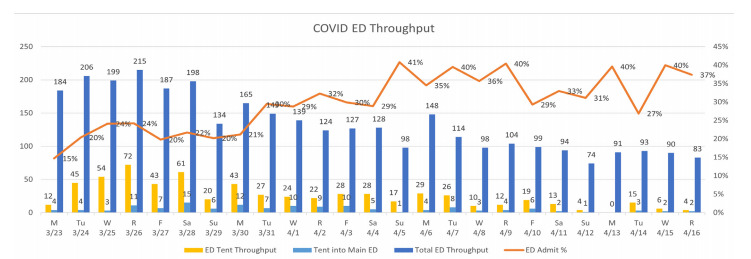
Patient throughput data.

The FTA processed 634 patients between March 23 and April 16, 2020 with a mean of 26.4 patients per day (SD 18.2, range 4–72) comprising 15–25% of overall adult ED volume during the study period. Of the 634 patients evaluated, 499 (79%) were discharged and 135 (21%) were transferred into the ED for further evaluation. Of the 135 brought into the ED, 81 (12.7% of the total) were admitted (Figure 5). Patients needing further evaluation were predominantly male: 67.4% (91/135) of the transfers into the ED and 77.8% (63/81) of the admissions. Patients transferred into the ED tended to have a higher pulse rate (105.4 vs 93.7 beats per minute) and respiratory rates (21.5 vs 18.1 breaths per minute), and lower oxygen saturation (93.9% vs 97.8%) (Table 1 and 2).

Of the 634 initially screened patients, 58 (9.1%) returned to the ED for re-evaluation. The average return after discharge was six days. Of the 58 patients who returned, 17 (29%) were admitted with their average time to return 3.5 days and an admission lasting on average 5.4 days. Of those admitted, two patients had notable outcomes:

1. Initial visit (temperature [T] 99.3°F; heart rate [HR] 94; respiratory rate [RR] not recorded; blood pressure [BP] 153/80 millimeters mercury (mm Hg); oxygen levels ]SpO_2_] 97%) 41-year-old male with a past medical history of hypertension and hyperlipidemia, returned the following day (T 97.8°F; HR 90; RR 28; BP 164/98 mm Hg; SpO_2_ 86%) complaining of shortness of breath, found to have acute renal injury and admitted. On hospital day two he was intubated for worsening hypoxia and on hospital day five experienced cardiac arrest without return of spontaneous circulation.
2. Initial visit (T 101.3 °F; HR 110; RR 16; BP 121/86 mm Hg; SpO2 96%) 40-year-old male with no medical history returned three days later (T 102.9 °F, HR 131; RR 20; BP 136/87 mm Hg; SpO_2_ 90%) and admitted for increased work of breathing. He was hospitalized for 14 days requiring supplemental O_2_ via non-rebreather mask and discharged home with no invasive intervention.

Tables 1 and 2 describe the association between age and initial vital signs (pulse, respiratory rate, oxygen saturation) with undergoing additional evaluation in the ED or being admitted. Age >= 70 years old (Odds Ratio (OR) [6.52], 95% Confidence Interval (CI), (1.40–30.38) p-value= 0.02), increased pulse (OR [1.03] (CI, 1.01–1.05) p-value=0.003), respiratory rate (OR [1.19] (CI, 1.08–1.30) p-value <0.001), and oxygen saturation (OR [0.60] (CI, 0.52–0.70) p-value <0.001) appear most correlated with the outcome of transfer into the main ED for evaluation. Of note, although gender appeared important in the descriptive data, the effect of gender was no longer significant in the final model when initial vital signs were included. The Hosmer-Lemeshow goodness of fit for the into-the-ED model was 0.61. With the larger the value, the better the model observed events align with the expected events. The model fit of 0.61 is a good fit for the data. For association with being admitted to the hospital, age >=70 years of age (OR [6.48] (CI, 0.99–42.48) p-value 0.05), respiratory rate (OR [1.23] (CI, 1.11–1.35) p-value <0.001) and oxygen saturation (OR [0.52] (CI, 0.44–0.62) p value <0.001). The Hosmer-Lemeshow goodness of fit for the admission to hospital model was 0.90.

Patient throughput averaged about thirty minutes and never more than one hour. The FTA closed early on 4/12/20 due to physician illness and closed completely on 4/13/20 due to dangerously high winds. The FTA operations ceased on 4/16/20 due to volume of throughput not substantiating the number of staff necessitated to keep the facility open.

## DISCUSSION

The use of this external, COVID-19-specific FTA allowed our ED to screen an average of 25 COVID-19 PUIs per day (up to >70/day at peak COVID-19 surge volume) with the ability to safely discharge 79% of those patients. This comprised 15–25% of overall adult ED volume during the time frame. The external structure screened patients rapidly and determined whether further medical evaluation in the main ED was needed and expedited rapid discharge with return precautions without needing to enter the main ED in most cases. The establishment of a FTA decreased the workload of the main ED staff and their interactions with infectious patients, during a time when available isolation and resuscitation rooms were already beyond capacity. In contrast, during pre-COVID-19 ED workflow, patients had contact with at minimum the following staff: greeter RN, greeter clerk, triage RN, PCA, ED RN, MD/PA/residents. The sequestering of a large cohort of well appearing, COVID-19 PUIs also allowed our fast-track area to be more available, and safer for patients with non-COVID complaints. Environmental cleaning services were delayed leading to excessive room closures.

By using this external structure, infectious pathogens were kept outside. Within this clear hot zone, staff members donned full PPE and were more compliant with appropriate precautions. The walkway up to the entrance area was at a slight incline, which may have allowed for a mild stress test before vitals were assessed – an unintentional design that identified patients for further evaluation who might otherwise have been overlooked.

The area had several lanes of workflow that functioned in concert allowing flexing up as volume increased with three simultaneous lanes working at the zenith of disease. Lanes were staffed by a variety of non-EM staff (floor nurse managers, dental residents, pediatric residents, oncology NPs, etc), which prevented diversion of ED resources already in short supply. The flow rate was further maximized by not performing COVID-19 testing as per NYC DOH policy.

The ED FTA appeared to be a highly functional model to effectively assess surges of ambulatory, COVID-19 PUI patients while keeping other ED patients and healthcare staff separate. With a discharge rate of 80%, only one significant adverse outcome resulted in death (0.16%), we believe this FTA was successful in its role and operations.

Certain variables (elevated pulse rate, elevated respiratory rate, decreased oxygen saturation) appeared to be associated with higher likelihood of needing further assessment in the ED. If these results are similar to those noted in other communities, these variables could be incorporated into screening pathways to triage patients to the main ED for further evaluation. Additional future screening pathways could predefine which patients would benefit from a telehealth follow-up visit.

## LIMITATIONS

The majority of the staff with the exception of the ED attending did not necessarily have emergency medicine experience. Limitations of this study included the individual bias of the greeter as no strict screening algorithms were used other than clinical impression. Due to the novel nature of COVID-19, during the study period there were no well validated clinical pathways for managing ambulatory COVID-19 patients, potentially resulting in some variability of practice patterns among providers and no ability to compare our efforts with any kind of standard practice or “gold standard.” While we attempted to follow up our patients and did have a 9.1% return rate, the current scope of this study did not allow us to assess how many patients had a negative outcome at home or sought care at another institution, although it should be noted that patients we did discharge from the forward screening area generally had minor symptoms and normal vital signs during a time when hospital and citywide systems were operating in a state of emergency, overwhelmed by the COVID-19 pandemic surge. As this was a single-center study, the concept may not be fully generalizable. Given the process was meant to be streamlined and minimalistic in scope, a treatment area like this could be implemented at other EDs. Further prospective evaluation would assess how an ED FTA can be optimized during similar surges.

Further research pertaining to the associated variables can be used in other field models and would be helpful. Aggregated prediction models are needed to identify which COVID-19 patients will have worse prognoses so that accurate clinical decision rules can be derived and validated. Our study was not designed to perform this work.

## CONCLUSION

Our ED forward treatment area was an effective method to rapidly screen the increased volume of patients with a novel infectious pathogen in an urban environment with limited resources. This treatment area decreased the burden on the ED structure, was rapidly deployed, and effectively screened patients for safe discharge home.

## Supplementary Information



## Figures and Tables

**Table 1:** Characteristics of forward treatment area patients discharged vs referred to the emergency department vs admitted.

	Discharged (n = 499)	Into ED (n = 135)	Admitted (n = 81)
Age category			
18–29 years old	88	9	4
30–39 years old	120	23	11
40–49 years old	134	35	19
50–59 years old	94	38	25
60–69 years old	48	22	17
≥70 years old	15	8	5
Gender			
Female	278	44	18
Male	271	91	63
Initial vital signs (mean [SD]; median [IQR])			
Pulse (n = 499)	94 (15.6); 94 (83–104)	105 (20.1); 105 (92–119)	107(16.1); 108 (92–119)
Respiratory rate (n = 304)	18 (2.2); 18 (16–20)	22 (5.6); 20 (18–24)	23 (6.2); 22 (18–25)
Temperature (°F) (n = 499)	99.0 (0.88); 98.8 (98.5–99.3)	99.1 (4.6); 99.1 (98.5–100.1)	99.1 (5.8); 99.3 (98.7–100.5)
Oxygen saturation (%) (n =497)	98 (1.5); 98 (97–99)	94 (4.8); 95 (92–97)	92.1 (5.2); 94 (90–96)
Systolic BP (mm Hg) (n = 498)	137 (19.0); 135 (124–149)	137 (20.8); 134 (122–151)	136 (21.5); 134 (120–151)
Diastolic BP (mm Hg) (n = 498)	84 (11.3); 83 (77–90)	82 (12.8); 81 (75–89)	82 (12.5); 81 (75–86)

*ED*, emergency department; *SD*, standard deviation; *IQR*, interquartile range; *F*, Fahrenheit; *BP*, blood pressure; *mm HG*, millimeters mercury,

**Table 2:** Logistic regression models for association characteristics with patients going into the emergency department) and being admitted.

	Into ED (OR (CI))	P-value	Admitted (OR (CI))	P-value
Age category				
18–29 years old (SD)	Reference		Reference	
30–39 years old (SD)	1.72 (0.59–5.03)	0.32	2.04 (0.47–8.94)	0.34
40–49 years old (SD)	2.28 (0.83–6.30)	0.11	1.97 (0.47–8.20)	0.35
50–59 years old (SD)	2.42 (0.86–6.76)	0.09	2.90 (0.71–12.00)	0.14
60–69 years old (SD)	1.35 (0.39–4.65)	0.87	2.23 (0.47–10.46)	0.31
>=70 years old (SD)	6.52 (1.40–30.38)	0.02	6.48 (0.99–42.48)	0.05
Initial Vital Signs				
Pulse	1.03 (1.01–1.05)	0.003	1.01 (0.98–1.03)	0.63
Respiratory rate	1.19 (1.08–1.30)	<0.001	1.23 (1.11–1.35)	<0.001
Oxygen saturation (%)	0.60 (0.52–0.70)	<0.001	0.52 (0.44–0.62)	<0.001
	Hosmer-Lemeshow chi^2^ = 0.61		Hosmer-Lemeshow chi^2^ = 0.90	

*ED*, emergency department; *OR*, odds ratio; *CI*, confidence interval; *SD*, standard deviation.
