# Supplementary material for: Impact of a Novel Emergency Department Forward Treatment Area During the New York City COVID-19 Surge
Source: West J Emerg Med. 2021 Jul 14;22(4):871–7. doi: 10.5811/westjem.2021.3.50653 (PMC8328161; doi:10.5811/westjem.2021.3.50653)
Supplement: Supplementary file 1 [file wjem-22-871-s001.docx]

Appendix 1

Just-In-Time Training

~0800 ED Wide Huddle whereas current information was disseminated to the entire ED – approximately 15 minutes

~0815 FTA Huddle – approximately 15 minutes

1. Review PPE
2. Review EPIC functionality
3. Review how to ask for help
4. Review goals, expectations

**Jacobi ED Tent Operations**

**Objective:**

1. To rapidly evaluate patients presenting to the emergency department with COVID like symptoms for severity of illness.
2. To evaluate these patients in a forward treating area to limit contagious illness from entering the emergency department unless medical intervention is necessitated.
3. To cohort contagious illness away from the emergency department staff in a safe area to allow rapid discharge of patients when clinically able.

**Processes:**

1. Arrive 8a to the Emergency Department North Side for Check-In
2. We will assist in getting you PPE from the ED command center; if you have goggles already, please bring them with you
3. Do not bring any personal items with you that cannot get contaminated in the tent (i.e. white coat, bags, etc). The tents are heated/Air Conditioned.
4. 830am we have a huddle in the North Side
5. Please coordinate your breaks so there are no overlaps to allow continued movement of patients, we are seeing 50+ patients currently and anticipate this number will increase to 100+ shortly.
